# What do we really know about addressing burnout among healthcare workers? Maybe less than we think

**DOI:** 10.1093/haschl/qxag041

**Published:** 2026-02-21

**Authors:** Ron Z Goetzel, Louis E Fazen, Karen B Kent, Enid C Roemer, Ian J Saldanha

**Affiliations:** Institute for Health and Productivity Studies, Johns Hopkins Bloomberg School of Public Health, Baltimore, MD 21205, United States; Department of Environmental Health and Engineering, Johns Hopkins Bloomberg School of Public Health, Baltimore, MD 21205, United States; Institute for Health and Productivity Studies, Johns Hopkins Bloomberg School of Public Health, Baltimore, MD 21205, United States; Institute for Health and Productivity Studies, Johns Hopkins Bloomberg School of Public Health, Baltimore, MD 21205, United States; Department of Epidemiology, Johns Hopkins Bloomberg School of Public Health, Baltimore, MD 21205, United States; Department of Health Policy and Management, Johns Hopkins Bloomberg School of Public Health, Baltimore, MD 21205, United States

**Keywords:** burnout, healthcare workers, systematic review

## Abstract

This policy inquiry responds to the question: Are commonly held assumptions about how to address healthcare workers' burnout truly evidence-based? We think not. Our conclusion is informed by findings from a recently completed large systematic review commissioned by the Agency for Healthcare Research and Quality. We highlight significant gaps in research regarding effective interventions directed at healthcare worker burnout. We include findings related to social support initiatives where limited credible evidence points to the potential for positive effects. We conclude that certainty about the effectiveness of intervention programs directed at burnout is elusive and there is a dire need for additional studies using rigorous methods given the healthcare worker shortage we now face.

Key Points

- Burnout among healthcare workers is a persistent concern because of its remarkable prevalence and potential impact on the health and well-being of medical providers and the delivery of quality care to patients.
- Most burnout interventions lack sufficient evidence and would benefit from more stringent research protocols.
- More intensive and sustained training interventions may be more likely to reduce burnout than sporadic or short-term interventions.

## Narrative

In early 2025, we published a paper entitled *Burnout Among Healthcare Workers: Unavoidable and Solvable*.^1^ The question posed in that paper was: Why are we not doing better at mitigating burnout in healthcare workers even though we know so much about the causes, consequences, and methods for addressing it?

Now, following a year-long systematic review of US healthcare worker (HCW) burnout conducted by the Johns Hopkins Evidence-based Practice Center and funded by the US Agency for Healthcare Research and Quality, we pose a new question: Are the commonly held assumptions about addressing healthcare worker burnout truly evidence-based?

Our systematic review uncovered significant gaps in the research regarding effective interventions, which led us to conclude that there is not sufficient evidence to support some long-held assumptions about addressing healthcare worker burnout.^2^ While the systematic review narrowly reported on the causes and consequences of healthcare worker burnout, and the degree of effectiveness and strength of evidence of organizational interventions targeting HCW burnout, this paper fills in the gaps to make sense of the scientific literature. Specifically, in this commentary, we present our own interpretation of what we do know, based on the identified evidence, and what is still up in the air and in need of additional rigorous research, especially as it relates to the most common forms of interventions.

## Why focus on burnout?

Burnout among healthcare workers is a persistent concern because of its remarkable prevalence and potential impact on the health and well-being of medical providers and the delivery of quality care to patients.

According to most recent studies, approximately half of physicians and nurses in the US report significant burnout symptoms in the form of high emotional exhaustion, high depersonalization, and/or a low sense of personal accomplishment.^3-5^ This burden appears to have peaked during the most demanding periods of the COVID-19 pandemic, although burnout in the healthcare field was already a long-standing issue before the pandemic.^6,7^

The potential impacts of burnout are substantial. Many studies point to consequences such as poor patient outcomes, low patient satisfaction, and increased employee turnover—factors that may further exacerbate healthcare delivery conditions and stressors for those workers who remain.^8^

## How we systematically reviewed healthcare worker burnout

In our systematic review, we sought to answer 3 key questions related to burnout of healthcare workers in the United States: (1) what are the causes, (2) what are the consequences, and (3) what interventions are effective in reducing burnout. This commentary focuses on our findings related to the third question about interventions.

To maximize the relevance of the evidence to today's context, we looked at studies from 2014 to 2025. To identify the most rigorous evidence that facilitated causal inference, we examined longitudinal study designs rather than ones that were simply cross-sectional. This meant excluding studies simply showing correlation between two co-existing phenomena. Instead, we aimed to uncover evidence of cause-and-effect relationships between independent variables (eg, occupational exposures and interventions) and subsequent dependent variables (eg, reduction in burnout).

Among longitudinal studies, we included several types of study designs, and we assessed the strength of evidence based on sample selection, attrition, observation period, statistical methods, and other factors. More rigorous research designs, such as randomized controlled trials that assigned healthcare workers to treatment (burnout prevention interventions) or control conditions (no intervention or another burnout prevention intervention), weighed more highly in our assessment of the evidence than pre–post studies without randomization or comparison groups.

Among interventions, we purposely focused on organizational solutions rather than ones directed at changing healthcare workers' psychosocial responses to stressors. Using an occupational health perspective, this approach was framed by the “hierarchy of controls” framework, whereby the most effective interventions *eliminate* occupational hazards rather than relying on individual initiative to increase personal resilience.^9^

We required a burnout measurement at baseline, prior to the intervention occurring, and then at the study's conclusion. Despite our narrow criteria, the considerable heterogeneity in the definition, measurement, analysis, and reporting of burnout outcomes made evidence synthesis challenging.

## What we found

Overall, the strength of evidence for most of our areas of inquiry was rated as *low or insufficient* due to such factors as study design limitations, small sample sizes, short observation periods, risk of bias, and inconsistency of results across studies. Regarding interventions, our overall conclusion was that most burnout interventions lacked sufficient evidence and would benefit from more stringent research protocols. That does not necessarily mean that the interventions themselves were ineffective; rather, the research supporting these programs did not show an effect. The preponderance of low-quality evidence does not exclude the possibility that many interventions may demonstrate positive effects with larger sample sizes and more rigorous protocols.

## Training and social support interventions to address healthcare worker burnout

We classified the organizational interventions assessed in the literature into 4 broad categories: training, social support, resource, and scheduling interventions. Of these, training and social support interventions are often the “go to” methods to reduce burnout given their low cost and minimized disruption to operations.

Nearly half (47%) of the 72 identified studies of interventions evaluated training programs. We examined diverse training interventions focused on increasing clinical knowledge and skills, patient communication, professional skills, technological skills, as well as permutations and combinations of these skill enhancement approaches. Most of the evidence we identified had insufficient strength to warrant a conclusion. Based on those findings, we concluded that trying to reduce burnout by offering additional training was unlikely to succeed.

About a quarter (26%) of the intervention studies focused on providing social support. These included peer, team, or supervisor support programs, such as facilitated peer group discussions, grief support, daily team huddles, recognition programs, and establishing formal communication mechanisms for employees to discuss issues with their supervisors. Among these 19 studies focused on social support interventions, we did find some bright spots.

The evidence from the systematic review suggested that it may be important for a social support program to be proactive, not reactive. This was because interventional studies with debriefings, triggered by specific negative events (eg, patient deaths), showed no significant effect on burnout.^10-12^

The evidence also confirmed that superficial interventions provided inconsistent results. For example, interventions that fostered collegiality or respect through daily team huddles^13^ or formal recognition programs were unlikely to be effective.^14-17^ By contrast, programs that fostered more intensive, sustained, and meaningful interpersonal connections horizontally among peers,^18-21^ as well as vertically with supervisors,^22^ produced more consistent positive effects.

Facilitated peer group discussions were shown to be a particularly promising form of social support interventions. Six studies, including 3 randomized control trials, examined these types of discussions and found that they decreased depersonalization among healthcare providers, with results often extending several months after the intervention.^18-20,23-25^

In one study published in 2014, West et al. conducted a randomized controlled trial that exposed 74 practicing physicians to a series of 19 biweekly facilitated physician discussion groups incorporating reflection and shared experience.^18^ Notably, this intervention provided protected time (1 hour of paid time every other week) for participants to engage in the discussion groups. Although no effects were observed during or at the conclusion of the intervention, measures taken at 3 and 12 months after the end of the study revealed statistically significant reductions in high depersonalization in the trial intervention arm.^18^

In a more recent randomized control trial, West et al. provided further evidence that self-facilitated physician small-group meetings may reduce depersonalization.^19^ This study randomized 125 practicing physicians to participate in the intervention (12 biweekly self-facilitated discussion groups involving reflection, shared experience, and small-group learning, over 6 months) or remain on a waitlist. Six months after completion of the intervention, the prevalence of high depersonalization decreased significantly by 11.4% in the intervention arm vs a 2.0% increase in the control arm.^19^ This study also demonstrated reductions in other measures of the Maslach Burnout Inventory, including overall burnout.

A third randomized controlled trial by Linzer et al. assessed regular monthly provider meetings to discuss work–life challenges, personal stressors, and complex patient care situations as part of a complex intervention among 135 clinicians. Although the authors did not measure depersonalization, they did report a significant difference in the proportion of clinicians with reduced burnout at least 1 year later.^21^

Similarly, in a trial of 151 healthcare workers, Furnari et al. found that those assigned to the facilitated group discussions experienced a significant decrease in depersonalization at 3 months relative to the control group with no intervention. Facilitated group discussions were also observed to reduce depersonalization in two pre–post studies among residents^25^ and a mixed group of physicians, nurse practitioners, and certified nurse midwives.^24^

Although promising, these results were often limited to depersonalization as an outcome, one of 3 components of burnout. Neither of the trials by West (2014) or Furnari, for example, found evidence of improvement in emotional exhaustion or overall burnout. Moreover, the effect was likely small, and not apparent when using continuous measures of burnout^19^ or on the initial analysis.^21^ A small effect size may also explain null findings in very small studies, such as a trial by Seto et al., as well as the fluctuating effects in less intense studies and shorter follow-up periods.

What is remarkable, however, is the durability of response that extends well beyond the intervention period. All studies that followed participants beyond 1 year, including 3 randomized controlled trials and one pre/post study, observed improvement at those longer time points. Very long-term effects, such as those reported by West,^18^ may be influenced by differential attrition, for example, those without burnout return more frequently to take the surveys at later time points.

Another possible explanation holds more promise. Facilitated peer-group discussions may serve as seeds for more substantive friendships and collegiality among colleagues and coworkers down the road. In this case, we would expect fewer results with less intense interventions and at earlier time points, and better results with more extended follow-up, as those seeds turn to roots and long-term relationships become self-sustaining well beyond the intervention period, which is precisely what we saw in our synthesis of the data.

Facilitated group discussions, which often take place after hours and involve sharing food outside of the workplace, may be less important than the general cultivation of meaningful interpersonal connections among coworkers. We did not examine purely psychosocial interventions, such as resident happy hours or more informal gatherings. However, there is some evidence to indicate that these encounters may be effective in reducing depersonalization. While such interventions may not reduce exposure to occupational hazards, they may soften the effects of day-to-day stressors experienced by healthcare workers and may therefore be effective over long-term horizons.

## The bottom line

Unfortunately, our systematic review found more evidence for the absence of effects than evidence supporting commonly cited interventions to reduce burnout. Most training interventions to improve clinical, technological, and communication skills had no significant impact on burnout. These training programs may require higher intensity and/or greater frequency to be effective.

Similarly, we found no consistent evidence that implementing peer support through daily care team huddles, grief support, or employee recognition programs reduced burnout.^10-12,14,15,25^ However, in general, we did notice that the more intensive and sustained interventions demonstrated the greatest benefit, suggesting that the dose for these interventions may be key to their effectiveness.

Although the uneven results of the systematic review remain open to interpretation, our optimistic view is that what may matter most are the opportunities to develop meaningful relationships with peers and colleagues in healthcare settings, the time horizon for the program (whether those interactions are one-time or ongoing), and the composition and the commitment of participating healthcare workers, all of which may not be easily replicable. Conversely, work conditions that impair the development of such relationships, such as employee turnover and irregular shifts, may be particularly damaging to the well-being of healthcare workers.

## Future research

Taken together, the existing evidence on healthcare worker training to prevent burnout suggests 3 fruitful avenues for future research. *First*, larger studies with more rigorous controlled designs are needed to detect small effects. *Second*, more intensive and sustained training interventions, such as 1 hour per week over an entire year, may be more likely to reduce burnout than sporadic or short-term interventions. *Third*, more comprehensive follow-up of participants and reporting of outcomes is needed to explore the heterogeneity and differential effects of interventions across burnout scales.

As for social support interventions, interventions that showed benefit were those with the most direct contact between employees and supervisors implemented for a full year of structured leadership engagement. Future research on supervisor support would benefit from exploring similar high-intensity and sustained contact between supervisors and employees. Further research is also needed on facilitated peer group discussions, especially when implemented more broadly across all employees in an occupation, specialty, or workgroup rather than simply among the most willing volunteers.

Finally, it is important to note that the literature we reviewed was primarily focused on nurses, physicians, and medical residents. We need more published research for other professions too, as their workflows, needs, and visibility by leadership are unique from those of physicians and nurses.

Training and social support interventions may reduce the occupational hazards of burnout. Rigorously designed research is needed to evaluate intense, sustained interventions that facilitate stronger human connections—among peers, and between supervisors and subordinates. This requires greater leadership commitment, and accompanying resources, to establish a workplace culture that continually promotes employee health, safety, and well-being.

## Supplementary Material

qxag041_Supplementary_Data
